# Lead-Induced Impairments in the Neural Processes Related to Working Memory Function

**DOI:** 10.1371/journal.pone.0105308

**Published:** 2014-08-20

**Authors:** Jeehye Seo, Byung-Kook Lee, Seong-Uk Jin, Jang Woo Park, Yang-Tae Kim, Hun-Kyu Ryeom, Jongmin Lee, Kyung Jin Suh, Suk Hwan Kim, Sin-Jae Park, Kyoung Sook Jeong, Jung-O Ham, Yangho Kim, Yongmin Chang

**Affiliations:** 1 Department of Medical & Biological Engineering, Kyungpook National University, Dong-In dong, Jung-gu, Daegu, Korea; 2 Korean Industrial Health Association, Seoul, Korea; 3 Department of Psychiatry, School of Medicine, Keimyung University, Daegu, Korea; 4 Department of Radiology, Kyungpook National University College of Medicine, Dong-In dong, Jung-gu, Daegu, Korea; 5 Department of Radiology, College of Medicine, Dongguk University, Gyeongju, Korea; 6 Department of Occupational and Environmental Medicine, Ulsan University Hospital, University of Ulsan College of Medicine, Ulsan, Korea; 7 Department of Psychiatry, Ulsan University Hospital, University of Ulsan College of Medicine, Ulsan, Korea; 8 Department of Occupational and Environmental Medicine, Dongguk University College of Medicine, Dongguk University Ilsan Hospital, Goyang, Korea; 9 Department of Occupational and Environmental Medicine, Soonchunhyan University Hospital, Cheonan, Korea; 10 Department of Molecular Medicine, Kyungpook National University College of Medicine, Dong-In dong, Jung-gu, Daegu, Korea; University Of São Paulo, Brazil

## Abstract

**Background:**

It is well known that lead exposure induces neurotoxic effects, which can result in a variety of neurocognitive dysfunction. Especially, occupational lead exposures in adults are associated with decreases in cognitive performance including working memory. Despite recent advances in human neuroimaging techniques, the neural correlates of lead-exposed cognitive impairment remain unclear. Therefore, this study was aimed to compare the neural activations in relation to working memory function between the lead-exposed subjects and healthy controls.

**Methodology/Principal Findings:**

Thirty-one lead-exposed subjects and 34 healthy subjects performed an n-back memory task during MRI scan. We performed fMRI using the 1-back and 2-back memory tasks differing in cognitive demand. Functional MRI data were analyzed using within- and between-group analysis. We found that the lead-exposed subjects showed poorer working memory performance during high memory loading task than the healthy subjects. In addition, between-group analyses revealed that the lead-exposed subjects showed reduced activation in the dorsolateral prefrontal cortex, ventrolateral prefrontal cortex, pre supplementary motor areas, and inferior parietal cortex.

**Conclusions/Significance:**

Our findings suggest that functional abnormalities in the frontoparietal working memory network might contribute to impairments in maintenance and manipulation of working memory in the lead-exposed subjects.

## Introduction

Occupational exposure during industrial processes to lead, which is a heavy metal, can have toxic effects on multiple organ systems including renal dysfunction, hematopoietic dysfunction, and reproductive dysfunction [1]–[5]. Moreover, lead has been shown to be a typical neurotoxicant in many occupations [6]. Although occupational exposure to this neurotoxicant has declined steadily over the past 20 years, its toxicity, especially its subclinical neurotoxicity is seen frequently in workers who are chronically exposed to it [7].

Lead-exposed workers often show impaired performance on neurobehavioral tests involving attention, processing speed, visuospatial abilities, working memory, and motor function [8]–[11]. It has also been revealed that lead can adversely affect general intellectual functioning [12]. Schwartz and colleagues have suggested that lead has a chronic effect on cognitive decline as a function of cumulative dose [6]. Similarly, chronic exposure to lead at higher concentrations has been shown to result in significant reductions in cognitive functions such as memory, learning, and verbal concept formation [13], [14].

Despite recent advances in human neuroimaging techniques, the neural correlates of lead-exposed cognitive impairments remain unclear. Accordingly, in the present study, we used functional magnetic resonance imaging (fMRI) to assess the neural correlates of lead-induced memory impairments in workers with subclinical dysfunction in working memory networks as a result of chronic exposure to lead. We hypothesized that subjects with lead exposure would show abnormal brain activity in the frontoparietal memory network compared to that of healthy subjects. Moreover, we assumed that memory deficits which are associated with an increased functional demand on higher memory load would be seen. Therefore, we performed fMRI and used the 1-back and 2-back memory tasks to investigate the behavioral significance of additionally recruited brain regions in retired workers with chronic lead exposure and control individuals.

## Methods

### 1. Subjects

A total of 65 volunteers (31 lead-exposures and 34 healthy controls) were recruited in this study. The subjects were age and sex-matched (60.4±5.5 years in the female lead-exposure group vs. 59.3±5.2 years in the female healthy controls) and all were right-handed. We recruited retired former female lead workers who had worked in plants producing lead batteries. Control subjects were female manual workers who were not exposed to lead, or solvents in other factories in the same geographic area in Korea. All subjects had normal vision and had Korean as a first language. Exclusion criteria for all subjects included: (a) a history of neurological condition, (b) a history of a medical condition associated with cognitive dysfunction, and (c) abnormal changes in the brain MRI. They all agreed to participate in our fMRI study and provided written informed consent. One hundred fifty dollar was provided to each participant for participation in the study, because they could not work due to several hours spent for examination and traffic. The protocol used for this study was approved by the local Internal Review Board.

### 2. Determination of lead in whole blood

Blood lead was measured in duplicate with a Zeeman background-corrected atomic absorption spectrophotometer (model Z-8100; Hitachi, Tokyo, Japan) using the standard addition method of the National Institute of Occupational Safety and Health. Blood samples were diluted 1∶10 for blood lead with 1% Triton X-100 in distilled water using 0.5% ammonium phosphate as a modifier, and 15-µl aliquots of the samples were injected onto the platform of the furnace [15].

All blood lead analyses were carried out by the Institute of Environmental and Occupational Medicine, Soonchunhyang University, a laboratory certified by the Korean Ministry of Labor. Since 1988, the institute has served as a reference laboratory for blood lead assessment in a Korean quality control and assurance program. It is licensed by the Ministry of Labor as a uniquely designated institute for nationwide occupational health services to lead industries. For the internal quality assurance and control program, commercial reference materials were obtained from Bio-Rad (Whole Blood Metals Control). The method detection limit for blood lead in the present study was 0.60 µg/dL. No sample was below the detection limit.

### 3. Clinical laboratories

Hemoglobin was determined using the cyanmethemoglobin method (Beckman Coulter Inc., model Ac-T 8, United States), and hematocrit was measured using the capillary centrifugation method [16]. Zinc protoporphyrin (ZPP) was measured by a hematofluorometer (Aviv, United States) [17]. The urinary aminolevulinic acid (ALA) levels were determined according to the method of Tomokuni et al [18]. Creatinine in urine was analyzed by the Sigma kit (St. Louis, Missouri, United States) [19].

### 4. Verbal working memory task

**Figure 1:**
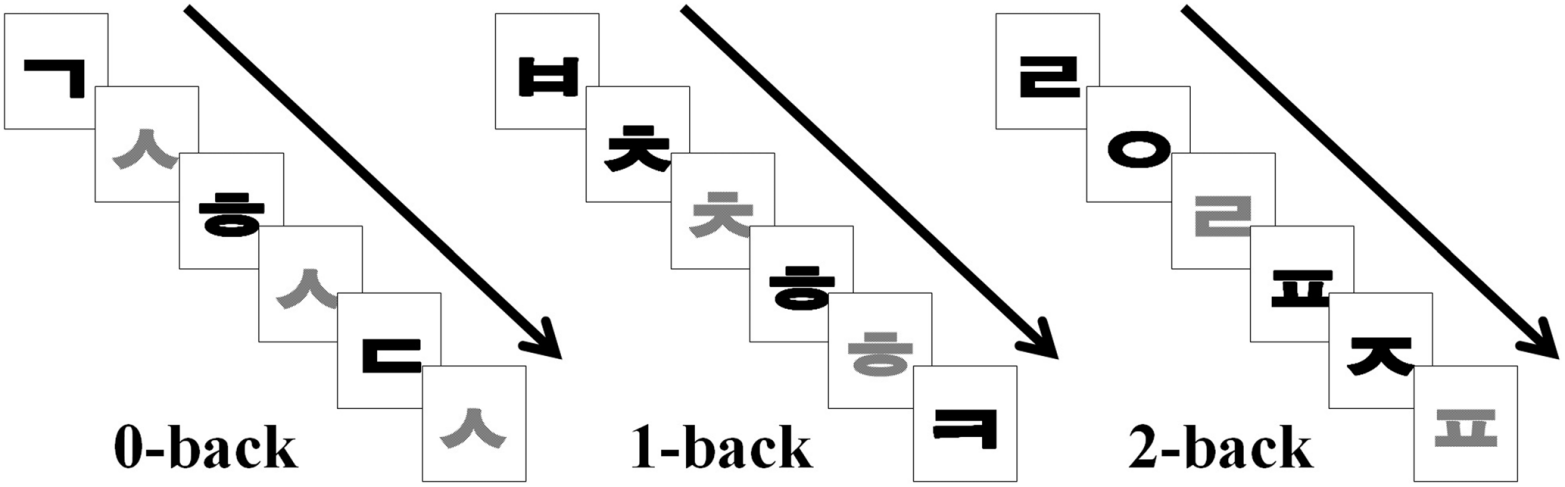
The N-back working memory paradigm. In the 0-back condition, participants were asked to press a button if a specific target letter “
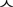
” appeared. In the N-back (N is 1 or 2) condition, they were asked to respond when a letter matched one that had been presented N letters before the present letter.

The working memory paradigm consisted of the N-back memory task (Figure 1). The N-back task, where N is an integer (usually 0, 1, 2, or 3, requires on-line monitoring, updating, and manipulation of remembered information, and is therefore assumed to place great demands on a number of key processes within working memory [20]. Participants performed a letter N-back task with two conditions: 0-back and N-back. In the 0-back condition, participants were asked to remember a target letter that was presented at the beginning of each trial block. In the N-back (N is 1 or 2) condition, they were asked to respond when a letter matched one that had been presented N letters before the present letter. We used letters from the Korean alphabet as target cues. Stimuli were displayed using SuperLab (Cedrus Corp., version 4.5, San Pedro, CA). When SuperLab detects the MRI scan trigger, it immediately starts the N-back stimulus task. The stimuli were presented binocularly using a goggle-based system (modified Silent Vision SV-7021 Fiber Optic Visual System, Avotec Inc., Stuart, FL) positioned on top of the head coil. Participants were asked to press a button with their right index finger if a specific target appeared. For example, in the 2-back task, participants determined whether an item was the same as that two trials back. If the item was the same, participants pressed the button under their right index finger. Participants pressed the button under their right middle finger if the item was different to that presented two trials back. To ensure that the participants understood the task demands, they rehearsed outside the scanner prior to the fMRI investigation, practicing a lettered 0- and N-back memory task that had the same stimulation timing as the subsequent fMRI paradigm. The experiment utilized a blocked design with two epochs for each of the two experimental conditions (4 epochs in total). Each stimulus letter was visible for 500 ms and was followed by a fixation cross that randomly appeared for 2500 or 3500 ms. Ten letters were presented in each epoch of trials, so that each epoch lasted 36 s. The probability of a letter being a target was 31%. The entire functional scanning run took approximately 4 min 48 sec.

### 5. Functional magnetic resonance imaging

Blood oxygenation level dependent (BOLD) contrast was collected for each subject using a 3.0 T GE EXCITE (Milwaukee, WI) scanner equipped with a transmit-receive body coil and a commercial eight-element head coil array. T2*-weighted echo planar imaging was used for fMRI acquisition. The following acquisition parameters were used in the fMRI protocol: echo time (TE) = 40 ms, repetition time (TR) = 3000 ms, field of view (FOV) = 22 cm, acquisition matrix = 64×64. Using a midsagittal scout image, 31 contiguous axial slices with 4 mm thickness were placed along the anterior-posterior commissure (AC-PC) plane covering the entire brain. A 3-dimensional T1-weighted anatomical scan was obtained for structural reference. The following acquisition parameters were used in the 30 dimensional T1-weighted fast spoiled gradient echo (FSPGR) for anatomical scan protocol: TE = 3 ms, TR = 7.8 ms, flip angle (FA) = 20°, acquisition matrix = 256×256.

### 6. Functional image analyses

Image processing and statistical analyses for fMRI data were carried out using MATLAB (The Mathworks Inc., Natick, MA) and SPM8 (SPM; Wellcome Department of Imaging Neuroscience, London, UK; online at http://www.fil.ion.ucl.ac.uk). Data were converted from DICOM to NIFTII format, processed using a slice timing correction with the first acquired slice to corrected for temporal offsets in the acquisition of slices and spatially realigned and unwarped to the first image for correction movement and distortion. The mean fMRI volume and FSPGR were coregistered using mutual information, and normalized to the Montreal Neurological Institute (MNI) brain [21]. The normalized data were smoothed with isotropic Gaussian kernel FWHM 8 mm.

The pre-processed fMRI data were then entered into first-level individual analysis by comparing fMRI activity during the N-back task with that during the 0-back (N-back>0-back). In second-level within-group analysis, contrast images from the analysis of individual subjects were analyzed by one-sample t-tests, thereby generating a random-effects model, allowing inference to the general population. The SPM{t}s were thresholded at P<0.05, family-wise error (FWE)-corrected for multiple comparisons across the whole brain. For comparison of neural activity between the lead-exposed subjects and controls group during 2-back memory task, contrast images for the main effects were assessed using a two-sample t-test. Spatial extent thresholds were determined by 2,000 Monte Carlo simulations using AlphaSim as implemented in the SPM REST toolbox [22]. Input parameters to AlphaSim included an individual voxel threshold probability of 0.005, cluster connection radius of 5 mm, and 8 mm FWHM smoothness. The estimated minimum cluster size extent was 52 voxels for two sample t-test map in order to satisfy an FWE rate correction of P<0.05.

Estimates of percent signal change during the n-back task were calculated from the activation and deactivation regions of each participant using the MarsBaR-software (http://marsbar.sourceforge.net) and ROIs defined by the Anatomical Automatic Labeling (AAL) ROI library [23]. The average signal used in this calculation was based on all conditions and identified as the beta value for the mean column of the regression analysis.

### 7. Statistical Analysis

We examined the univariate distributions of the continuous variables to determine their normality. To decrease skewness, some variables were transformed to their natural logarithms and presented as geometric means (GM) and range; otherwise, arithmetic means (AM) and standard deviations (SD) were used. Mean values of continuous variables were compared using the Student’s *t*-test. Differences in the proportion of workers who smoked or educational level were determined using the chi-square test. The difference in BOLD signal change of activated brain regions between the two groups was examined with the Student’s *t*-test. Pearson correlation analyses were used to determine the correlations between mean percentage changes in BOLD fMRI signal in the activation brain regions and working memory performance (response accuracy) in individual subjects. We assessed the effects of blood lead on percent signal change by multiple regression analysis. To select an appropriate multiple regression model, we first analyzed the possible interaction of educational level on the association between brain signal changes and blood lead concentrations using a single model with educational level×blood lead concentrations interaction terms in multiple regressions after adjusting for covariates, and we found that educational level did not interact. Collinearity was not observed between educational level and blood lead concentration. All statistical analyses were performed using SPSS software. Statistical significance was defined at *P*<0.05.

## Results

### 1. General characteristics

The demographic, clinical, and laboratory characteristics of the 31 lead-exposed workers and 34 control individuals are listed in Table 1. All of the participants were women, and there was no significant age difference between the lead-exposed workers and the control workers. The educational levels of the lead-exposed workers were significantly lower than those of the controls (P<0.05). There were no significant differences between the groups in terms of smoking status, hemoglobin levels, hematocrit levels, ZPP levels, or urinary ALA levels. Most of women were non-drinker, without difference between two groups (data not shown). The mean blood lead concentration was significantly higher in the lead-exposed workers than in the control group. Among the lead-exposed workers, the duration of lead exposure was 8.5 (1.4–20.7) years, and the time since the cessation of exposure was 11.9 (4.9–21.0) years. Lead-exposed workers showed higher blood lead levels than the control group, even though they had not been exposed to lead for a long time.

**Table 1:** Demographic, clinical, and laboratory characteristics of study subjects.

	Lead-exposed group	Control group	Statistics
	n = 31	n = 34	p-value
Age (year)	60.4±5.5	59.3±5.2	0.425
Smoking, n (%)			0.477
Current smoker	0 (0.0%)	0 (0.0%)	
Ex-smoker	1 (3.2%)	0 (0.0%)	
Non-smoker	31 (96.8%)	34 (100.0%)	
Educational level n (%)			**<0.05**
Elementary school or less	24 (77.4%)	15 (44.1%)	
Middle school	5 (16.1%)	10 (29.4%)	
High school or more	2 (6.5%)	9 (26.5%)	
Duration of exposure (year)*	8.5 (1.4∼20.7)		
Years since cessation of exposure*	11.9 (4.9∼21.0)		
Blood lead level (µg/dL)*	4.07 (0.88∼13.5)	2.00 (1.24∼6.47)	**<0.001**
Zinc protoporphyrin*	62.1 (41∼86)	58.1 (28∼139)	0.309
Urinary ALA*	2.20 (0.54∼4.30)	2.14 (0.40∼4.56)	0.832
Hematocrit (%)	39.1±2.5	38.5±2.6	0.312
Hemoglobin (g/dL)	13.0±0.9	12.8±0.9	0.509
Task performance			
Response time (msec)			
1-back	1441.4±562.3	1227.6±371.5	0.073
2-back	1309.6±521.1	1157.5±365.4	0.175
Task accuracy (%)			
1-back	55.9±19.8	65.4±19.4	0.056
2-back	61.4±20.1	77.2±15.6	**0.001**

Mean ± SD, *:GM (range); in this case statistical significance was tested with log-transformed variables.

### 2. N-back Task Performance

In terms of accuracy and response time, the mean performance on the N-back tasks was inferior in the lead-exposed group compared to that of the control group (Table 1). The differences in task accuracy between the two groups were statistically significant (*P*<0.01), except for 1-back accuracy.

### 3. fMRI data

**Figure 2:**
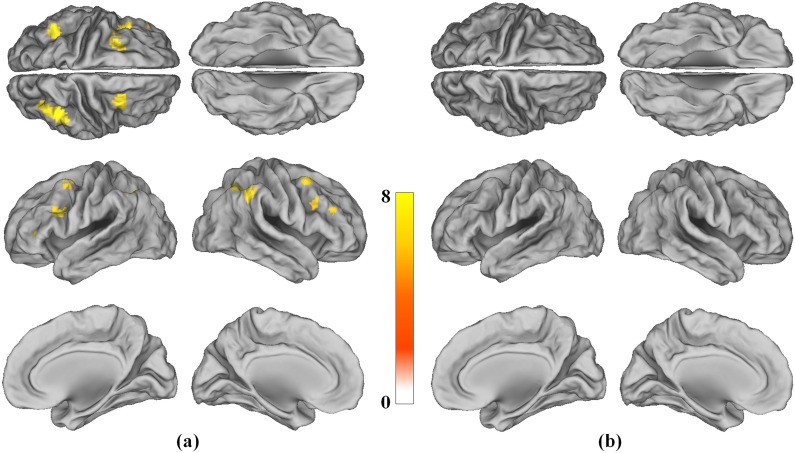
Within group analysis of 2-back task. One-sample t-test group comparison in the control group (a) and in the lead-exposed subject group (b) (P<0.05, FWE-corrected for multiple comparison).

**Figure 3:**
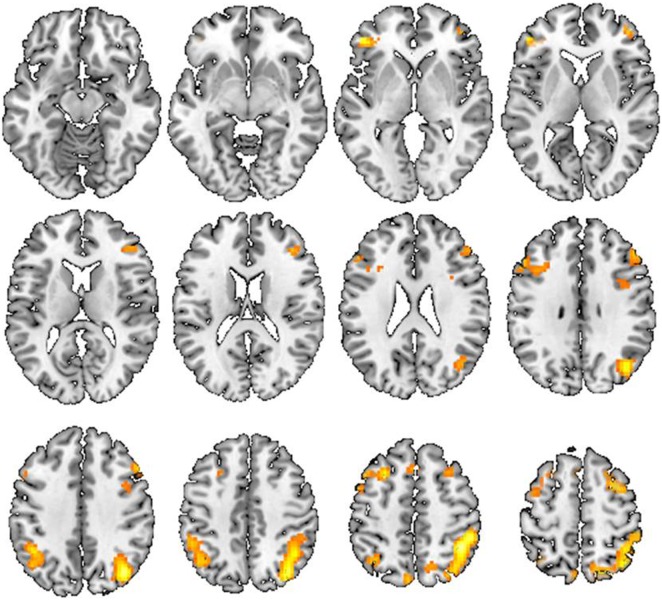
Between group analysis of 2-back task. The brain areas, which activated in control subjects compared to the lead-exposed subjects from between group analysis of 2-back task. Statistics correspond to an FWE rate correction of P<0.05 estimated using Monte Carlo simulations.

To evaluate the possible confounding effects of education, all group analyses were performed with educational level as a covariate. The within-group analyses, which were thresholded at P<0.05 and family-wise error (FWE)-corrected for multiple comparisons across the whole brain, showed activity in the network of the frontal and parietal cortical areas in the healthy control group, while no regions showed significant activity in the lead-exposed subject group, for the 2-back working memory task (Figure 2). The activation network included the ventrolateral prefrontal cortex (VLPFC), inferior parietal cortex (IPC), dorsolateral prefrontal cortex (DLPFC), medial and lateral premotor cortex (BA6 or pre-SMA), and the inferior frontal cortex (IFC) (Table 2). Direct comparisons between the groups showed that, during the 2-back working memory task, the healthy control group showed higher activation than the lead-exposed subject group in the bilateral DLPFC and IPC (Figure 3 and Table 3). No region showed significantly higher activation in the lead-exposed subject group compared to the healthy control group. No region showed significant activity in either the lead-exposed or control groups for the 1-back working memory task.

**Table 2:** Regions of activation from within-group analysis during 2-back task at P<0.05, FWE-corrected for multiple comparisons in control group.

			Coordinates			
	Side	Cluster size	x	y	z	T
Dorsolateral prefrontal cortex	L	274	–33	7	55	7.92
	R	168	33	8	55	7.05
Pre-supplementary motor area	C	107	0	20	49	7.20
Ventrolateral prefrontal cortex	L	34	–45	41	4	6.85
Inferior frontal cortex	L	136	–45	16	26	7.82
	R	133	45	37	24	6.82
Inferior parietal cortex	L	216	–36	–58	52	8.36
	R	216	41	–55	52	7.23

C = center, L = left, R = right.

**Table 3:** Regions of activation in control subjects compared to the lead-exposed subjects from between-group analysis during 2-back task at P<0.05, FWE-corrected for multiple comparisons.

			Coordinates			
	Side	Cluster size	x	y	z	T
Dorsolateral prefrontal cortex	L	106	–39	2	55	3.67
	R	97	38	8	61	3.75
pre-supplementary motor areas	C	24	–2	23	52	3.28
Ventrolateral prefrontal cortex	L	49	–42	38	1	3.97
	R	36	42	36	13	3.48
Inferior frontal cortex	L	74	–42	17	28	3.05
	R	31	39	5	34	3.05
Orbito-frontal cortex	L	30	–45	41	–5	3.10
	R	28	42	50	4	3.43
Inferior parietal cortex	L	174	–36	–58	53	3.60
	R	179	42	–49	49	4.52

C = center, L = left, R = right.

### 4. Mean percentage changes in BOLD fMRI signals

**Figure 4:**
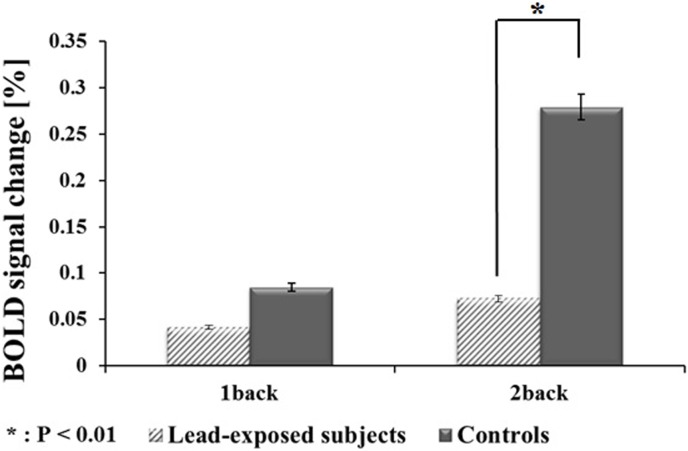
BOLD signal changes for N-back working memory task performance. The difference in 2-back task accuracy between lead-exposed subjects (oblique line) and control subjects was statistically significant (P<0.05, Student’s t-test), except for 1-back task accuracy.

The mean percentage changes in the BOLD signals in each group in the activated networks showed that, only for the 2-back task, the control group had significantly stronger BOLD activity than did the lead-exposed subject group (Figure 4).

Table 4 lists the results of multiple regression analysis of the BOLD signal changes using log blood lead level as a predictor. Age and educational level, which are important factors in the BOLD signal changes, were included as covariates. After controlling for age and educational level, blood lead level was inversely associated with the BOLD signal changes in left DLPFC, right IPC, and right IFC. Educational level was also positively associated with signal changes.

**Table 4:** Multiple regression analyses for BOLD signal changes with log blood lead concentration as a predictor.

Brain areas	Beta coefficients (95% CI)			Model	
	Log blood lead	Educational level	Age	R^2^	P-value
Left DLPFC	–0.125 (–0.232∼–0.018)*	0.032 (0.005∼0.059)*	–0.003 (–0.06∼0.010)	0.184	0.002
Right DLPFC	–0.120 (–0.241∼0.0001)	0.048 (0.018∼0.079)*	–0.001 (–0.015∼0.013)	0.224	<0.001
Right IPC	–0.094 (–0.172∼–0.015)*	0.028 (0.008∼0.047)*	0.0001 (–0.010∼0.009)	0.214	0.001
Right IFC	–0.087 (–0.152∼–0.022)*	0.021 (0.004∼0.037)*	–0.004 (–0.011∼0.004)	0.236	<0.001

* P<0.05.

**Figure 5:**
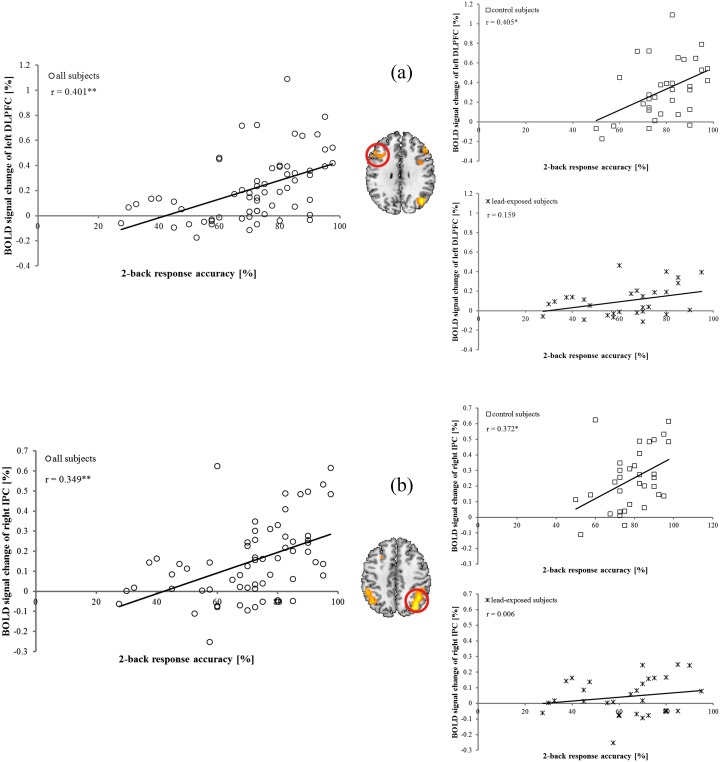
Correlation between BOLD signal change and 2-back task performance (response accuracy). Percentage BOLD signal change in the left DLPFC (a) and right IPC (b) showed positive correlation with response accuracy in control subjects, while such trend was no longer showed in lead-exposed subjects (* : P<0.05, ** : P<0.01).

We found a positive trend for an association between the percentage changes in the BOLD signals in the networks activated in the 2-back task and the response accuracy for all subjects (Figure 5). For the control subjects, we found such a trend for several brain regions. For the lead-exposed subjects, however, such a trend was no longer found for any of the activated brain areas.

## Discussion

The goal of the present study was to use fMRI to elucidate the neural differences between retired lead-exposed and healthy subjects involved in a working memory task. This is the first study to describe the neural correlates of working memory networks in retired lead-exposed workers. To date, no previous studies have used the verbal N-back test to compare brain activation patterns in occupational lead-exposed and healthy subjects.

The GM (4.07 µg/dL) of workers with past exposure to lead is higher than that (2.00 µg/dL) in control group, and the latter is similar to the GM (2.01 µg/dL) of female general population in Korea [24]. The study subjects (ex-workers) ceased exposure to lead for approximately 10 years, and blood lead concentrations indicate recent exposure to lead [25]. Thus the GM (4.07 µg/dL) of blood lead concentrations suggest high body burden of lead during a past life.

In the between-group analysis of 2-back, lead-exposed subjects showed reduced activity in distributed cortical networks that mediate executive function, particularly in the prefrontal and parietal areas, compared to healthy subjects. BOLD signal changes in these areas, left DLPFC, right IPC and IFC, in particular, were associated with blood lead level after controlling for age and educational level, showing dose-response relationship between lead exposure and brain activation. Thus brain activations are probably due to past exposure to lead.

During performance of the 2-back task, the prefrontal cortex is thought to mediate the monitoring of a series of stimuli, adjusting the information that is held in working memory to incorporate the most recently presented stimulus, while rejecting more temporally distant stimuli [20]. This result is in line with prior studies that have demonstrated abnormalities in prefrontal cortex in lead-exposed subjects [26], [27]. Lead-exposed children have shown reductions in the *N* acetylaspartate to creatine and phosphocreatine ratios in the frontal gray matter, suggesting increased neuronal loss [26]. Young adults with greater lead exposure in early childhood had lower activation in distributed cortical networks, especially those including the left frontal cortex, during the performance of a verb generation task [27]. Therefore, our findings lend further evidence that lead can induce neurotoxic effects in prefrontal areas. Within the prefrontal cortex, lead-exposed subjects showed reduced activation in the DLPFC and VLPFC compared to healthy subjects during the 2-back task. With regard to models of cognitive control, the DLPFC maintains context in order to provide a task-appropriate response [28]. In addition, the VLPFC is concerned specifically with remembering or retrieving information during the implementation of an intended act or plan [20]. Therefore, the functional abnormalities of the prefrontal cortex in lead-exposed subjects could be associated with impairments in the maintenance and manipulation of working memory.

Additionally, we found that lead-exposed subjects had lower activation in the inferior and superior parietal cortexes compared to healthy subjects during the 2-back task. Whereas the dorsal IPC plays an important role in retaining information temporally and switching attention rapidly, the ventral IPC has been shown to be associated with phonological encoding and recording processes [29]. Furthermore, it is well known that the parietal cortex may support domain-general executive processes [30] and that activation in the posterior parietal cortex is associated with sustaining attention [31] and task switching [32], especially in the right hemisphere. Consistent with these studies, our data showed right hemisphere dominance of attention-related activity in the posterior parietal cortex in healthy controls compared to subjects with lead exposure when performing the 2-back task.

Moreover, the lead-exposed subjects exhibited significantly lower activation of the pre-SMA and significantly worse performance on the 2-back task compared to controls. The pre-SMA has been shown to be involved in language generation [33], maintaining working memory [34], representing action intentions [35], and switching between action sets [36], [37].

In the present study, differences in 2-back task accuracy between the two groups were statistically significant. We also found a positive correlation between response accuracy on the 2-back task and brain activation in the frontal and parietal cortical regions in the control subjects, suggesting that individuals performing with high accuracy needed more neural resources than individuals performing with low accuracy. However, such a positive correlation between response accuracy and brain activation in the frontal and parietal cortical regions was not shown in the lead-exposed subjects. This suggested that, due to neuronal deficits in the frontal and parietal cortical regions, the neural activation in the lead-exposed subjects approached a ceiling for the 2-back task. Despite this neural ceiling effect, we found group differences in the response accuracy on the 2-back task between the control and lead-exposed subjects. This suggested that we need a future study to draw firmer conclusions regarding the behavioral and neural performances of lead-exposed subjects on 2-back tasks.

The present study has a toxicological implication that a long time after lead exposure (11.9 years), some memory defects persist [38], [39]. Past lead exposure can have a long term neurotoxic effect, notably because of a delayed release from bones [39], [40].

Our study has some limitations. First limitation is our lack of information about past exposure to lead. Study subjects of the present study are ex-workers exposed to lead. Blood lead concentrations which show recent exposure to lead do not reflect total body burden of lead in ex-workers. The lack of previous information of lead exposure may influence the further interpretation especially in aged persons. Further study of bone lead with X-ray fluorescence which reflect longer-term cumulative exposures will be needed [25]. The study on active lead-exposed workers is also needed to be done compared to retired workers. In addition, there is a lack of information about past medical history such as silent infarction, hypertension, diabetes, hyperlipidemia. Second, the present cross-sectional study showed an association between brain activation and lead exposure; however, we cannot establish a causal relation with cross-sectional design. An unknown third factor might be a common link responsible for an observed association. A prospective study will be needed to confirm a causal relation. Third, the number of the study subjects that were recruited was rather small to have a firm conclusion, leading to an insignificant association in brain signal changes. Subgroup analyses with stratification of exposure level or educational level cannot be performed due to small sample size. Instead, we adjusted for educational level in multiple regression models. Finally, information on environmental lead exposure of subjects, such as water, air, or old house or building was not available. Two groups of subjects came from the same geographic area in Korea, and lead based-paint was not used in homes in Korean culture. Thus environmental lead exposure was considered to be similar between two groups. Taken together, further study is warranted to investigate the working memory networks not only in the retired workers but also in the current workers with large sample size.

In summary, the working memory networks of the retired lead-exposed workers showed significantly less activity in the frontoparietal memory network, as well as reduced working memory performance, compared to control subjects. These results suggested that the working memory deficits that were found in the retired lead-exposed subjects may be attributable to differences in the neural activation of the frontoparietal memory network and may result from neurotoxicant effect.
